# Patient Perceptions of Care Coordination during Neoadjuvant Therapy for Gastrointestinal Cancers: A Mixed Methods Analysis

**DOI:** 10.1007/s12029-024-01030-w

**Published:** 2024-02-14

**Authors:** Natalie M. Bath, Marilly Palettas, Lena Stevens, Angela Sarna, Aslam Ejaz, Alex Kim, Timothy M. Pawlik, Jordan M. Cloyd

**Affiliations:** Department of Surgery, Division of Surgical Oncology, The Ohio State University Wexner Medical Center, 410 W 10th Ave, N-907 Doan Hall, Columbus, OH 43210 USA

**Keywords:** Cancer care coordination, Neoadjuvant therapy, Gastrointestinal cancer, Mixed-methods analysis

## Abstract

**Purpose:**

Effective cancer care coordination (CCC) is an integral component of health care delivery and critical to achieving optimal oncologic outcomes. Neoadjuvant therapy (NT), the delivery of multimodality therapy prior to surgery, is inherently complex and multidisciplinary, but CCC during NT is poorly understood. The objective of this study was to characterize patient perceptions of CCC during NT using a mixed methods approach.

**Methods:**

This study is a cross-sectional analysis of patients with gastrointestinal cancers receiving NT who participated in a prospective longitudinal cohort study evaluating their real-time experience using a customized smartphone application. Patients completed the Cancer Care Coordination Questionnaire for Patients (CCCQ-P), a 20-item validated measure of care coordination quality, six weeks after initiating NT. Items were scored on a 5-point Likert scale, and subsections on communication (13 questions) and navigation (7 questions) were calculated with higher scores signifying better CCC. Univariate linear regression was used to calculate the impact of fragmented care and other factors on perceived CCC. Semi-structured interviews were conducted among a convenience sample of patients (*n* = 5); transcribed interviews were then coded using an inductive approach.

**Results:**

Among 82 participants, mean age was 61 years old, 68% were male, and mean number of comorbidities was 1.68. Overall (mean 76.6 out of 100), communication subsection (48.6 out of 65), and navigation subsection (28.0 out of 35) CCCQ-P scores suggested overall positive perceptions of care coordination. Qualitative analysis of patient interviews highlighted the need for coordination among physicians before communicating the plan to patients as well as the importance of providers communicating plans in verbal and written form.

**Conclusions:**

Successful completion of NT requires significant care coordination between patients and healthcare professionals. Yet, in this cross-sectional analysis of patients on a prospective cohort study, patient perceptions of CCC during NT were overall positive. Future research should focus on optimizing other aspects of care delivery in order to improve outcomes of NT.

## Introduction

Over the past 3 decades, outcomes for patients with gastrointestinal (GI) and hepatopancreatobiliary (HPB) malignancies have significantly improved, which is largely attributable to the development of more effective multimodality therapies. There has also been a shift in the timing of chemotherapy or radiation therapy delivery from immediately following surgery, known as adjuvant therapy, to preoperatively, known as neoadjuvant therapy (NT) [1]. Depending on cancer type, stage, and individual patient characteristics, advantages of NT may include the early treatment of micrometastatic disease, improved multimodality therapy completions rates, increased margin-negative resection rates, and the opportunity to test both individual tumor biology as well as in vivo effectiveness of the therapy [2–6]. Consequently, NT is increasingly used prior to surgery for most localized GI and HPB cancers [1].

While these trends in multimodality treatment have resulted in improved patient outcomes, the increased care complexity has led to new challenges in care coordination that may represent barriers to ensuring optimal outcomes for individual patients. Indeed, effective cancer care coordination (CCC) is an integral component of health care delivery and necessary for achieving oncologic outcomes [7]. Care coordination, broadly speaking, has been defined by the Agency for Healthcare Research and Quality (AHRQ) as the “deliberate organization of patient care activities between two or more participants involved in a patient’s care to facilitate the appropriate delivery of health care services” [8]. Previous research in the palliative setting has focused on investigating the quality of CCC and identifying factors associated with poor care coordination [9, 10].

NT is inherently multidisciplinary and requires patients to navigate a complex system that involves medical, nursing, and allied health professionals. Patients must juggle multiple appointments with medical, radiation, and surgical oncologists as well as numerous visits for imaging, laboratory tests, infusions, and referrals to other providers, all while experiencing the side effects of aggressive cancer treatment, side effects of the tumor left in situ*,* and uncertainty of whether surgical resection will occur in the future. Indeed, patients experience significant treatment burden during NT [11]. This complexity of care and treatment burden on patients also significantly impacts caregivers [12]. Given this complexity, patients are at increased risk of receiving poorly coordinated care which may interfere with their completion of intended therapy and achieving curative-intent surgery [9, 13]. Despite these realities, there is little existing literature on the quality of CCC during NT. Therefore, the objective of this study was to characterize patient perceptions of CCC during NT using a mixed methods approach and evaluate factors associated with poor CCC.

## Methods

### Study Design and Population

This study is part of a prospective longitudinal cohort study evaluating the patient experience during NT using a customized smartphone application. The complete protocol of this prospective study has previously been reported [14, 15]. In brief, enrolled patients with esophageal, gastric, pancreatic, hepatobiliary, colorectal, or bladder cancer between 2020 and 2021 were instructed on how to use the application in order to document real-time symptoms and also receive monthly quality of life surveys directly through their application. As part of the study, patients were also administered a one-time survey on care coordination six weeks after initiating NT. Inclusion criteria consisted of age ≥ 18 years old, access to a smartphone or computer with Internet connection, and English language speaking. NT could be delivered at either the investigators’ institution or a local institution. The Ohio State University Wexner Medical Center has a large comprehensive cancer center that includes multidisciplinary oncology providers, advanced care providers, patient care resource managers, nurse navigators, social workers, and other ancillary staff members; however, the available cancer care resources available at local treating institutions was unknown.

### Survey Tools

The Cancer Care Coordination Questionnaire for Patients (CCCQ-P), a validated measure of care coordination quality, was used for the study. CCCQ-P was originally developed from a cohort of Australian patients who were recently diagnosed with cancer to assess the impact of CCC on a patient’s experience. Ultimately, the objective of the questionnaire was to identify accurate measures of cancer care coordination with the goal of quality improvement [16]. It has since been used primarily for patients with gastrointestinal cancers and more recently, lung cancer [9, 10, 17]. CCCQ-P contains 20 items divided into sections on navigation and communication. Items were scored on a 5-point Likert scale and subsections on communication (13 questions) and navigation (7 questions) were calculated with higher scores signifying better CCC. Data on demographics, comorbidities, and details of NT including severe complications were collected on all enrolled patients. Severe complications were defined as hospital admission, infection/sepsis, neutropenia, as well as cardiovascular or renal toxicity requiring intervention.

### Interview Guide and Process

Semi-structured interviews were conducted using a convenience sample (*n* = 5) of patients during NT to further explore patient experiences with CCC. The interview script was developed using preliminary survey results, evidence synthesis, and expert opinion. Questions focused on specific aspects of receiving NT treatment that have been especially challenging, including coordination among physicians, coordination with staff, and communication with patients were included. Questions were open-ended and allowed for additional questions depending on the responses of the interviewees. Interviews were conducted over the phone, audio recorded, and then manually transcribed.

### Data Analysis

Demographic and clinical characteristics were summarized using descriptive statistics. Mean and standard deviations or median and interquartile range were used for continuous variables and frequencies and proportions for categorical variables. Standardized scores for care coordination were calculated from the responses of the CCCQ-P questionnaire, and scores were summarized by subsection using descriptive statistics and presented as means and standard deviations. Likert plots were also used to visually summarize responses from the CCCQ-P questionnaire. All analyses and graphs were generated in R version 4.1.

Qualitative analysis followed an integrated approach, including both an inductive and deductive coding methodology [18]. Two independent researchers read all interview transcripts and developed an initial framework using preliminary codes before a more in-depth, inductive coding process took place. Afterwards, all interviews were coded for sub-themes, and the codebook was iteratively updated until all interviews were analyzed. These qualitative results were then integrated with the quantitative results to elucidate a more complete description of care coordination during NT. The study was approved by our Institutional Review Board.

## Results

### Patient Characteristics

Between 2020 and 2021, 82 patients completed the CCCQ-P questionnaire during NT and are included in this study. Data regarding patient demographics, travel distance for NT, comorbidities, cancer type, complications, and details regarding surgery and NT length are reported in Table 1. The mean age was 61 years old, and 68% were male. Most patients (95%) were white, and 63% were married or with a partner. The majority of patients (85%) had 1 or 2 comorbidities. The most common cancer types included colorectal (44%), pancreatic (29%), and esophagus (16%). Most patients (59%) received NT at our institution. Among surveyed participants, 80% eventually went on to undergo surgery with 72% of those receiving surgery at our institution. The median length of NT was 3.3 months, and the median travel distance to our institution was 48 miles (Table 1).

**Table 1:** Demographic, clinical, and treatment characteristics of participants

Characteristic	***N *= 82 (%)**
**Age, *mean (SD)***	61 (11)
**Gender, *% (N)***	
- Male	56 (68)
- Female	26 (32)
**Race, *% (N)***	
- White	78 (95)
- Black	4 (5)
**Marital status**	
- Married/partner	52 (63)
- Single	14 (17)
- Divorced	9 (11)
- Widowed	5 (6.1)
- Other	2 (2.4)
**Employment status**	
- Retired	**39 (47.6)**
- Working	**27 (32.9)**
- Unemployed	**10 (12.2)**
- Disabled	**6 (7.3)**
**Comorbidities, *mean (SD)***	1.68 (0.72)
- 0	0 (0)
-1	38 (46)
-2	32 (39)
-3	12 (15)
**Smoker**	
- Never	34 (41)
- Former	35 (43)
- Current	13 (16)
**Cancer type**	
- Colorectal	36 (44)
- Pancreas	24 (29)
- Esophagus	13 (16)
- Gastric	5 (6.1)
- Liver	2 (2.4)
- Other	2 (2.4)
**NT at OSU**	
- Completed	48 (59)
- No	31 (38)
- Partially completed	3 (3.7)
**Severe complications during NT**	
- No	52 (79)
- Yes	14 (21)
**Surgery at OSU**	
- Yes	53 (72)
- No	15 (20)
- Outside facility	6 (8.1)
**NT**	
- Treatment length, *months*	3.3
- Distance travelled, *miles*	48

### Quantitative Results

**Fig. 1:**
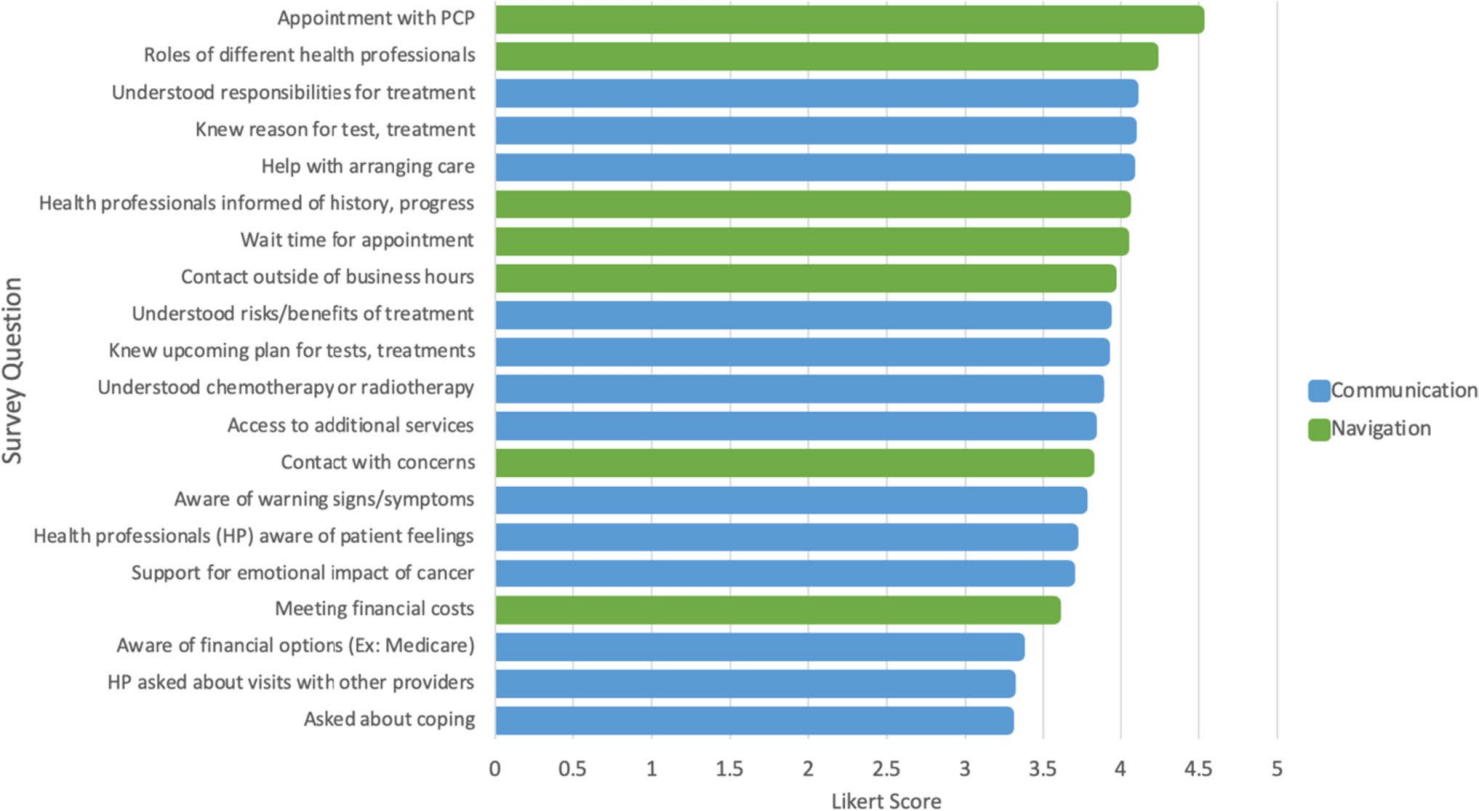
Cancer Care Coordination Questionnaire for Patients (CCCQ-P) results

Among all patients, the mean overall CCCQ-P score was 76.6 ± 11. Mean sub-section scores on communication (out of 65) and navigation (out of 35) were 48.6 ± 8 and 28 ± 4.3, respectively. Figure 1 demonstrates individual item responses ranked in order of best to worst. Aspects of care coordination regarding navigation rated highest by patients included ability to schedule appointment with primary care provider (4.5 ± 0.9) and understanding the roles of different health professionals (4.2 ± 0.9), whereas items ranked lowest included meeting financial costs (3.6 ± 1.3) and knowing who to contact with concerns (3.8 ± 0.9). With regard to communication within care coordination, aspects rated highest by patients included patient understanding responsibilities for treatment (4.1 ± 0.8) and the reason for tests and treatments (4.1 ± 0.8), whereas items ranked lowest included whether providers asked how they were coping with treatment (3.3 ± 1.1) and how visits with other providers were going (3.3 ± 1.1). Finally, on a scale of 1–10, perceived care coordination and quality of care was rated as 8.48 and 8.99, respectively.

On univariate analysis, no clinical or demographic factors including fragmented care (*p* > 0.05) were associated with patient-perceived care coordination (Table 2). On logistic regression, overall (1.02 (95% CI 0.98-1.07)), communication subsection (1.02 (95% CI 0.96-1.09)), and navigation subsection (1.06 (0.94-1.2)) CCCQ-P scores were not associated with likelihood of undergoing surgical resection.

**Table 2:** Univariate linear regression of factors associated with overall CCCQ-P scores

	**Estimate**	***P*-value**
Age	− 0.006	0.957
Gender (Ref = Female)		
- Male	− 0.629	0.818
Race		
- White vs Black (ref)	4.34	0.463
Marital status (Ref = divorced)		
- Married/partner	3.759	0.366
- Single	5.063	0.303
- Widowed	7.378	0.251
- Other/na	21.778	0.075
Travel distance	0.017	0.675
Comorbidities	− 0.363	0.839
Smoking Status (Ref = current)		
- Former	− 2.521	0.498
- Never	1.837	0.623
Cancer type (Ref = colorectal)		
- Esophagus	− 6.603	0.079
- Gastric	− 5.833	0.29
- Liver	− 8.833	0.293
- Pancreas	− 2.333	0.443
- Other	− 6.333	0.45
Complications (Ref = no)		
- Yes	− 0.1538	0.965
NT at OSU (Ref = no)		
- Yes	1.148	0.657

### Qualitative Responses

Qualitative analysis of participant interviews identified important themes of coordination among providers, coordination with staff, communication with patients, and other recommendations for enhanced CCC. Representative quotes for each theme are shown in Table 3.

**Table 3:** Qualitative results of care coordination during neoadjuvant therapy

**Representative quote by theme**
***Coordination among physicians***
*They sit down as a team. It was communicated to me that before my treatment starts, they sat down as a team and formulated a plan for me and then that plan was actioned. And then they did a great job at coordinating each stage.* *When I would see a new surgeon or specialist, they already knew about me. So, I knew they were communicating pretty good.*
*I’m not sure how that fell through the cracks, maybe it didn’t, maybe they were just hoping to hold off…*
***Coordination with staff***
*There was a question…and they took care of it. They got on it, called the insurance company and got the pre-approval process done. They have been pretty good about that.* *I didn’t check on my prescription refill…soon enough and I had to call them just to make sure they gave my pharmacy a call to make sure it was on order. […] But they took care of it over the phone. That was great.*
***Communication with patient***
*Each of the individual specialists, I can contact them. I got my patient care provider. And then also, when you go to visits, the nurses, when they give you the paperwork, there is always a number. When I leave any appointment, I have a printout sheet of what was talked about at the appointment, any follow-ups I need, and at the top of that is a contact number.* *I think everybody’s done very well informing me about what’s going on. They give me paperwork after every appointment, which explains everything, and they explain everything to me.* *I missed one appointment at one time because they didn’t call me to tell me when the appointment was until the day after.*
***Recommendations***
*Communication between my providers, if it is just a passing phone call, or a conversation at the hospital itself, it would be kind of nice if they let me know when they spoke and what they spoke of. […] It would be kind of handy if it were some kind of an update put on My Chart that they communicated with the other doctor and what they relate to each other.* *They do such a good job. It’s a resource I think you could expand on or get more out of by making them personal and saying: Susan is going to be your go to girl. So that you could almost call that person whenever and then maybe if Susan is not available because it’s her day off, or she is asleep, then she will say ‘I get back to you at this time. But if you need something in the meantime you can call your backup.’ So maybe you got Susan as number one. Tony is her backup. So, you have always got that as part of your team.*

In general, patients reported that coordination among physicians was an important aspect during NT. As one patient reported, “…It was communicated to me that before my treatment starts, they sat down as a team and formulate a plan for me and then that plan was actioned. And then they did a great job at coordinating each stage.” Patients particularly prioritized physicians coordinating among each other before relaying the plan to them. For example, one patient mentioned, “…it would be kind of nice if [providers] let me know when they spoke and what they spoke of.” Coordination with staff also had a significant impact on patients’ experience during NT. Examples included coordinating with insurance companies or pharmacies and facilitating appointments.

With regard to communication, participants emphasized the importance of providers communicating with patients both in verbal and paper form. As one patient stated, “When I leave any appointment, I have a printout sheet of what was talked about at the appointment, any follow-ups I need, and at the top of that is a contact number.”

## Discussion

Successful completion of NT requires the expertise and treatment coordination of medical oncologists, surgeons, radiation oncologists, and other care providers [11]. Consequently, the complexity of cancer care delivery has significantly increased as NT has become more widely adopted. In this cross-sectional study of patients with GI cancers receiving NT and using an established validated measure of CCC, we noted that patients had overall positive perceptions of care coordination. Consistent with survey results, qualitative responses were generally positive and emphasized the importance of understanding roles of each health professional and health professionals being informed of patient’s individual history and progress. On the other hand, few clinical factors were found to be associated with poor CCC scores suggesting the need for further research on optimizing care coordination and delivery during NT.

Effective care coordination is not only essential for the successful delivery of cancer treatment, but good coordination has previously been found to improve patient experiences during treatment [13, 19]. As a result of increased care complexity, patients with cancer frequently experience poorly organized and fragmented care [20]. Improvements in CCC were previously difficult to achieve since there was no accurate or reliable measure to monitor progress. Consequently, the development of the CCCQ-P survey was developed with the goal of assessing patient experience during treatment [16, 21]. CCC has been frequently studied with interventions being made in patient navigation, care delivery, and nurse case management, which have resulted in improved outcomes, patient experience, and end-of-life care [22, 23].

Although care coordination has been extensively studied in oncology, no studies to date have examined the impact of CCC in the neoadjuvant setting. CCC during the neoadjuvant period is particularly important since patients must deal with the emotional toll and physical side effects of their existing cancer and the uncertainty of whether they will be able to undergo surgery [24]. In our study, patient perceptions of CCC were generally positive which may be at least in part due to the copious resources available at our comprehensive cancer center. However, several areas of possible improvement were identified in both the quantitative and qualitative results. Items rated lowest by respondents included whether providers asked about their coping with treatment or visits with other providers. These findings demonstrate the importance of including discussion of these concerns during patient encounters. Patients also preferred receiving information in both verbal and written form [25]. We had hypothesized that fragmented care might be associated with CCCQ-P scores, but interestingly, no clinical, demographic, or treatment related factors were associated with quality of CCC suggesting the need for more research on optimizing care coordination.

Optimizing care delivery during NT is important not only for improving patient experiences and quality of life, but also in order to optimize completion of intended neoadjuvant treatment and receipt of surgical resection. Unfortunately, attrition during NT is common and associated with worse outcomes [26–28]. In a single institution study of patients with localized PDAC who started NT, Fong et al. found that locally advanced disease, decrease in performance status, and increase in CA 19–9 were among factors independently associated with failure to undergo surgical exploration [29]. At the same time, Kronenfeld et al. found insurance status to be the only factor associated with failure to undergo surgery in the gastric cancer setting [26]. Although we had hypothesized that poor care coordination may lead to increased adverse events and attrition during NT, interestingly, CCCQ-P scores were not associated with the likelihood of undergoing surgery. Nevertheless, additional research is needed to identify patient-, provider-, and process-related factors associated with attrition during NT.

Although the above findings are noteworthy, several limitations should be acknowledged. First, our cohort included patients with similar demographics who were treated at a single tertiary cancer center; therefore, our findings may not be applicable to more diverse populations. Specifically, patients of lower socioeconomic status, those primarily receiving care in the community, and those who are non-English speaking were not well represented in our study and yet may be particularly at risk for fractured care coordination. Second, participants in this study were limited to those who had smartphone access and thus may be more engaged in their healthcare compared to those without smartphones. Third, this cross-sectional analysis describes care coordination at a single point in time, and it is unknown whether quality of care coordination changes throughout the course of NT. Similarly, the study enrollment began at initiation of NT and therefore may not have explicitly evaluated care coordination during the diagnosis and evaluation phase. Finally, this study was designed and conducted during the COVID-19 pandemic; whether patient-reported experiences with care coordination were influenced by health care delivery changes as a result of the pandemic warrants further investigation.

In conclusion, patient perceptions of CCC during NT were overall positive despite the significant care coordination required during treatment. Few predictors of poor CCC were identified, and no difference was found in care coordination scores between patients who underwent surgery and those who did not. Additional research aimed at studying cancer care delivery in minority and non-English speaking patients, not well represented in the current study, will be important as this patient population may be particularly vulnerable to poor CCC. Additionally, future studies will focus on interventions to help improve of the quality care coordination with the goal of increasing completion of multimodal therapy and to improve patient quality of life during NT.
